# Copper homeostasis and cuproptosis in gynecological cancers

**DOI:** 10.3389/fcell.2024.1459183

**Published:** 2024-09-25

**Authors:** Xiaodi Huang, Mengyi Lian, Changzhong Li

**Affiliations:** ^1^ Center of Obstetrics and Gynecology, Peking University Shenzhen Hospital, Shenzhen, China; ^2^ Institute of Obstetrics and Gynecology, Shenzhen PKU-HKUST Medical Center, Shenzhen, China; ^3^ Shenzhen Key Laboratory on Technology for Early Diagnosis of Major Gynecologic Diseases, Shenzhen, China; ^4^ Department of Obstetrics and Gynecology, Longquan People’s Hospital, Lishui, China

**Keywords:** copper homeostasis, cuproptosis, gynecological cancers, prognosis prediction, functional analysis, copper ionophore

## Abstract

Copper (Cu) is an essential trace element involved in a variety of biological processes, such as antioxidant defense, mitochondrial respiration, and bio-compound synthesis. In recent years, a novel theory called cuproptosis has emerged to explain how Cu induces programmed cell death. Cu targets lipoylated enzymes in the tricarboxylic acid cycle and subsequently triggers the oligomerization of lipoylated dihydrolipoamide S-acetyltransferase, leading to the loss of Fe–S clusters and induction of heat shock protein 70. Gynecological malignancies including cervical cancer, ovarian cancer and uterine corpus endometrial carcinoma significantly impact women’s quality of life and even pose a threat to their lives. Excessive Cu can promote cancer progression by enhancing tumor growth, proliferation, angiogenesis and metastasis through multiple signaling pathways. However, there are few studies investigating gynecological cancers in relation to cuproptosis. Therefore, this review discusses Cu homeostasis and cuproptosis while exploring the potential use of cuproptosis for prognosis prediction as well as its implications in the progression and treatment of gynecological cancers. Additionally, we explore the application of Cu ionophore therapy in treating gynecological malignancies.

## 1 Introduction

As an essential trace element, copper (Cu) plays a pivotal role in various biological processes, encompassing antioxidant defense, mitochondrial respiration, and bio-compound synthesis (Chen et al., 2022). Within the human body, Cu maintains a delicate equilibrium and achieves homeostasis. Furthermore, the accumulation of Cu is associated with the manifestation of Wilson disease, a severe hereditary disorder (Walshe, 2007). Recently elucidated by Tsvetkov et al. (2022) in March 2022, cuproptosis represents a novel mechanism of cell death induced by excessive Cu ions. This phenomenon is distinct from other forms of programmed cell death such as apoptosis, ferroptosis, and necroptosis and relies on mitochondrial respiration. Specifically targeting lipoylated enzymes within the tricarboxylic acid (TCA) cycle primarily through Cu+ ions binding to them leads to subsequent oligomerization of lipoylated dihydrolipoamide S-acetyltransferase (DLAT), loss of Fe-S clusters and induction of heat shock protein 70 (HSP70), ultimately culminating in acute proteotoxic stress-induced cellular demise.

Gynecological cancers, including cervical cancer (CC), ovarian cancer (OC), and uterine corpus endometrial carcinoma (UCEC), significantly impact women’s quality of life and pose a threat to their survival. According to the Global Cancer Statistics 2020 report, CC ranks as the fourth leading cause of cancer-related deaths and is among the top four most commonly diagnosed cancers in women across all populations (Sung et al., 2021). OC, particularly epithelial ovarian cancer, stands out as the most lethal gynecological malignancy with 5-year survival rates below 45%. The lack of effective early diagnostic methods results in over 70% of OC cases being detected at an advanced stage (Webb and Jordan, 2017). UCEC represents a prevalent gynecological malignancy originating from the endometrium (Yang et al., 2020). Despite achieving a cure rate of up to 95% for early-stage UCEC cases, its prognosis remains poor due to high recurrence and mortality rates, causing over 50,000 deaths annually (Holst et al., 2019; Wang et al., 2020; Cui et al., 2021). It is evident that the detrimental impact of gynecological cancers on women’s health cannot be overlooked.

In recent years, numerous reports have emerged regarding the correlation between elevated levels of Cu and cancer progression. Evidence has demonstrated that many types of cancer exhibit increased intratumoral copper levels and/or altered systemic distribution of copper; for instance, in the early 1980s, significantly higher levels of Cu were found in malignant tissues within female reproductive organs (Margalioth et al., 1983; Denoyer et al., 2015). Excessive amounts of Cu are believed to play a crucial role in promoting cell proliferation, angiogenesis, and metastasis during cancer progression (Garber, 2015). However, few studies have investigated the association between gynecological cancers and cuproptosis, especially the effect of cuproptosis on the progression and treatment of gynecological cancers. This review not only discusses Cu homeostasis and cuproptosis mechanisms, but also explored the prospects for using cuproptosis as a prognostic tool for predicting outcomes in gynecological cancers, the role played by cuproptosis during gynecological cancer progression processes, as well as potential applications for Cu ionophores treatment. This review provides new insights into the application of coproptosis in gynecological cancers.

## 2 Copper homeostasis and cuproptosis

### 2.1 Copper homeostasis

After the ingestion of Cu-containing food and water, the majority of dietary Cu is absorbed in the small intestine by the copper transporter 1 (CTR1), a homotrimeric Cu transporter belonging to the solute carrier 31 (SLC31, CTR) class of proteins (Bossak et al., 2018). Prior to absorption by CTR1, Cu^2+^ in food is initially reduced to Cu+ at the apical membrane of enterocytes, most likely facilitated by Steap proteins which are a family of metalloreductases (Lönnerdal, 2008). Additionally, it has been suggested that divalent metal transporter 1 (DMT1), a major iron transporter, may also transport Cu at the apical membrane of enterocytes (Gunshin et al., 1997). Following absorption, Cu ions are exported from enterocytes into the bloodstream via ATPase copper transporting alpha (ATP7A) - one type of Cu-transporting ATPase (Cu-ATPase). Cu-ATPases are polytopic membrane proteins that regulate transmembrane transport of Cu ions through ATP hydrolysis energy utilization; this includes ATP7A and ATPase copper transporting beta (ATP7B) (Lutsenko et al., 2007). Cu ions primarily circulate in blood bound to various proteins. In human blood serum, approximately 75% of Cu^2+^ ions bind nonexchangeably with ceruloplasmin; among exchangeable Cu ions: about 25% bind with human serum albumin, while around 0.2% exist as ternary complexes comprising Cu^2+^-His-Xaa moieties (Kirsipuu et al., 2020). Subsequently, Cu is transported to the liver which plays a crucial role in regulating systemic copper homeostasis as it serves as a major captor and distributor while facilitating excretion via bile ducts (Li, 2020). The liver’s storage function for Cu is mediated by two thiol-rich proteins known as metallothionein1/2, capable of binding copper ions through their cysteine residues in pH-dependent manner (Xie et al., 2023). Furthermore, the liver can eliminate excesses amounts of Cu into the bile ducts through the function of ATP7B and release Cu into the circulation to redistribute Cu throughout the body (Hernandez et al., 2008). Ultimately, Cu is primarily excreted from the body through biliary secretion and unabsorbed metal ions, with a minor fraction being eliminated via urine, sweat, and menstruation (Aggett, 1999; Chen et al., 2022).

**FIGURE 1:**
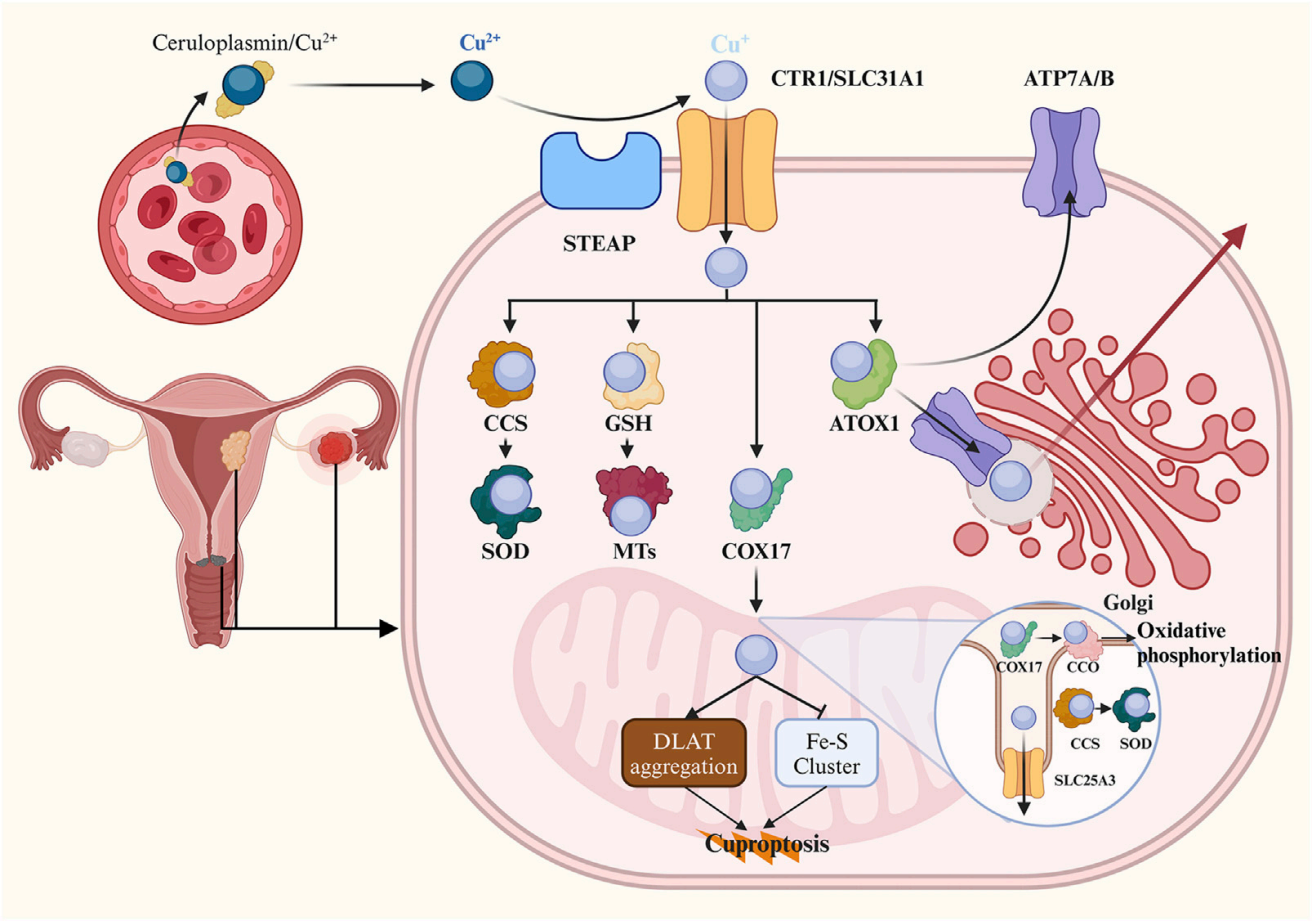
Schematic of copper homeostasis and cuproptosis. The majority of Cu^2+^ ions are transported by binding to ceruloplasmin in human blood serum. Cells uptake Cu+ ions through the Steap proteins and CTR1. Cellular Cu ions can bind to GSH and are then delivered to metallothioneins for storage in the form of a Cu-GSH complex. Cu can also target SOD1 through CCS to detoxify ROS. In the secretory pathway, Cu ions bind to ATOX1 and are delivered to ATP7A and ATP7B, which are involved in delivering Cu to the biosynthetic pathway and exporting it from the cell by targeting Cu-ATPases to different cellular membranes. Additionally, Cu is transported to CCO within mitochondria for oxidative phosphorylation and mitochondrial function via COX17. Within mitochondria, it is proposed that Cu directly binds to lipoylated proteins DLAT leading to lipoylation-dependent oligomerization in the TCA cycle. Furthermore, copper-induced programmed cell death occurs as a result of its ability induce loss of Fe-S cluster proteins in an FDX1-dependent manner. CTR1, Cu transporter 1; GSH, glutathione; SOD1, superoxide dismutase 1; CCS, copper chaperone for superoxide dismutase; ROS, reactive oxygen species; ATOX1, antioxidant-1 protein; ATP7A, ATPase copper transporting alpha; ATP7B, ATPase copper transporting beta; Cu-ATPase, Cu-transporting ATPase; CCO, cytochrome c oxidase; COX17, cytochrome oxidase 17 protein; DLAT, dihydrolipoamide S-acetyltransferase; TCA, tricarboxylic acid; FDX1, ferredoxin 1.

After cellular uptake, monovalent Cu ions are transported into cells via the Steap proteins and CTR1. Subsequently, Cu+ is either sequestered by metallothioneins (MTs) for storage or conveyed within the cytoplasm by copper chaperones. During the storage process, Cu ions initially bind to glutathione (GSH), and then Cu+ is delivered to MTs in the form of a Cu-GSH complex (Harris, 2000). Simultaneously, a precise regulatory network of high-affinity copper chaperones facilitates trafficking of non-stored Cu ions. Cu is directed towards superoxide dismutase 1 (SOD1) through its dedicated chaperone called copper chaperone for superoxide dismutase (CCS), which aids in detoxifying reactive oxygen species (ROS) and maintaining copper homeostasis. Animal experiments have demonstrated that both CCS and SOD1 are found in both the cytoplasm and mitochondrial intermembrane space, where they play a role in scavenging superoxides (Okado-Matsumoto and Fridovich, 2001; Sturtz et al., 2001). Copper ions are most likely transferred to antioxidant-1 protein (ATOX1), which acts as an intermediary for delivering Cu+ to ATP7A and ATP7B within the secretory pathway. These two transporters play crucial roles in supplying Cu for biosynthetic processes while also exporting excess intracellular Cu from cells through targeted localization on different cellular membranes (Lutsenko et al., 2007). In addition to transferring Cu to secretory compartments and cytosolic proteins, Cu+ is transported into mitochondria via cytochrome oxidase 17 protein (COX17), a specific chaperone for delivery to cytochrome c oxidase (CCO) involved in oxidative phosphorylation and mitochondrial function. Furthermore, within the cell nucleus, Cu+ can regulate CTR1 expression through Sp1 zinc finger domain acting as a sensor for monitoring intracellular Cu levels (Li, 2020). Figure 1 illustrates cellular mechanisms governing Cu homeostasis.

### 2.2 Cuproptosis

In the early 1980s, Cu was reported to induce cell death (Halliwell and Gutteridge, 1984). Over subsequent decades, there has been ongoing debate regarding the mechanism of programmed cell death associated with excessive Cu levels. However, in 2022, a novel mechanism called cuproptosis was elucidated and validated as the process by which Cu induce programmed cell death, distinguishing it from apoptosis, ferroptosis, pyroptosis, and necroptosis (Tsvetkov et al., 2022). Specifically, it was discovered that increased intracellular Cu levels resulting from pulsed treatment with the Cu ionophore elesclomol at concentrations as low as 40 nM could trigger cuproptosis. Importantly, pharmacological inhibitors known to block various cell death pathways were found to be ineffective in suppressing Cu-induced cell death. These inhibitors included necrostatin-1 (a necroptosis inhibitor), ferrostatin-1 (a ferroptosis inhibitor), N-acetyl cysteine (an inhibitor of oxidative stress-induced death), Z-VAD-FMK and Boc-D-FMK (apoptosis inhibitors). Conversely, only a Cu chelator demonstrated the ability to rescue cells from excess Cu-induced cell death. This finding highlights that cuproptosis is distinct from all other known forms of programmed cell death.

The mitochondrion is a crucial site for the biological role of Cu within cells and represents a primary target in cuproptosis. Excessive Cu ions are known to induce oxidative damage to the mitochondrial membrane and disrupt enzyme function in the TCA cycle (Sheline and Choi, 2004; Arciello et al., 2005). Previous studies have demonstrated that Cu impact the mitochondrial respiratory chain, resulting in elevated levels of intracellular ROS (Jiao et al., 2016; Kwan et al., 2016). In 2022, Tsvetkov et al. elucidated the strong association between cuproptosis and the TCA cycle rather than the electron transport chain or ATP production. They observed time-dependent dysregulation of numerous metabolites associated with the TCA cycle following pulse treatment with elesclomol. Conversely, treatment with elesclomol did not significantly reduce basal or ATP-linked respiration, while mitochondrial uncoupler had no effect on Cu toxicity (Tsvetkov et al., 2022). Subsequently, they identified and validated critical cuproptosis key genes (CKGs) through genome-wide knockout screens, metabolism screens, and individual gene knockout studies. Knockout of seven CKGs was found to rescue cuproptosis. Among these genes, Ferredoxin 1 (FDX1) encodes a reductase that converts Cu^2+^ to Cu^+^, thereby enhancing copper toxicity by directly binding to elesclomol-Cu complex and inhibiting Fe-S cluster formation (Tsvetkov et al., 2019). The remaining six genes can be divided into two groups: lipolytransferase1 (LIPT1), lipoyl synthase (LIAS), dihydrolipoamide dehydrogenase (DLD), which encode components of lipoic acid pathway; pyruvate dehydrogenase (PDH) complex including DLAT, pyruvate dehydrogenase E1 subunit alpha 1(PDHA1), pyruvate dehydrogenase E1 subunit beta (PDHB), which encode protein targets for lipoylation. Through immunohistochemical staining and gene knockout experiments, FDX1 was identified as an upstream regulator of protein lipoylation, a highly conserved lysine posttranslational modification found in only four enzymes involved in metabolic complexes within the TCA cycle (Rowland et al., 2018; Solmonson and DeBerardinis, 2018). It has been proposed that Cu directly binds to lipoylated proteins in the TCA cycle, leading to a toxic gain of function (Tsvetkov et al., 2022). DLAT, an essential component of the PDH complex, undergoes lipoylation-dependent oligomerization upon binding Cu ions. Additionally, Tsvetkov et al. (2022) also discovered that treatment with copper ionophore induces loss of Fe-S cluster proteins in an FDX1-dependent manner and triggers acute proteotoxic stress characterized by excessive HSP70 expression. The mechanism underlying cuproptosis reveals the interplay between mitochondrial respiration, the TCA cycle, and the Cu-induced programmed cell death (Figure 1).

## 3 Excessive Cu and cancer progression

Numerous studies have consistently reported elevated levels of Cu in both serum and tumor tissues across various types of cancers, including gynecologic malignancies (Gupte and Mumper, 2009). For instance, patients with CC exhibited significantly higher serum Cu levels compared to healthy individuals (Zhang et al., 2018), while cancerous ovary tissues showed a greater concentration of Cu than noncancerous ovary samples (Yaman et al., 2007). Besides, it has been reported that chronic exposure to elevated levels of Cu in drinking water can stimulate the proliferation of tumor cells and pancreatic cancer growth in mice by regulating the oxidative phosphorylation process (Ishida et al., 2013). These results suggest that excess Cu may be associated with the development and progression of cancers.

Recent studies have demonstrated that excessive Cu can participate in multiple signaling pathways, thereby promoting cancer progression by enhancing tumor growth, proliferation, angiogenesis, and metastasis. Here we summarized several pivotal signaling pathways implicated in the progression of cancer. In terms of cell proliferation, mitogen-activated protein kinase 1/2 (MEK1/2) was the first-identified copper-binding kinase belonging to the RAS/RAF/MEK/ERK pathway, which is believed to be involved in cell proliferation and cancer progression (Chang et al., 2003; Montagut and Settleman, 2009; Vitaliti et al., 2022). Copper ions can also act on the PI3K-AKT signaling pathway leading to downstream AKT activation through direct activation or binding to histidine 117 and histidine 203 sites of pyruvate dehydrogenase kinase 1 (Ostrakhovitch et al., 2002; Guo et al., 2021). Subsequently, AKT activation further catalyzes the phosphorylation and subcellular redistribution of forkhead box O1a and forkhead box O4, resulting in cancer cell proliferation (Walter et al., 2006). Additionally, ATOX1, one of the Cu chaperones, has been documented to augment cellular proliferation as a Cu-dependent transcription factor while promoting inflammatory neovascularization (Itoh et al., 2008; Chen et al., 2015).

Angiogenesis is another crucial step in tumor progression with excess Cu considered an essential co-factor for angiogenesis (Tisato et al., 2010). The role of Cu in the regulation of blood vessel formation has been demonstrated through its mediation of angiogenin’s biological activity, promotion of microvessel invasion and vascular infiltration, as well as upregulation of vascular endothelial growth factor (VEGF) expression level (Soncin et al., 1997; Gérard et al., 2010; Qiu et al., 2012). Hypoxia inducible factors (HIFs) play a crucial role in tumor angiogenesis by activating the transcription of VEGF, angiopoietin 2, stromal-derived factor 1, cyclooxygenase 2, and stem cell factor (Jun et al., 2017). Importantly, Cu is essential for HIF-1 activation through its regulation of the binding between HIF-1α and the hypoxia-responsive element as well as the formation of the HIF-1 transcriptional complex (Feng et al., 2009).

Moreover, some studies have demonstrated that Cu can exert its effects on various proteins and pathways to promote tumor invasion and metastasis. Cu has been shown to activate lysyl oxidase/lysyl oxidase like proteins secreted by cancer cells, thereby promoting tumor metastasis through remodeling of the tumor microenvironment and induction of epithelial-mesenchymal transition (EMT), a critical process in cancer cell invasion (Xiao and Ge, 2012; Vitaliti et al., 2022). Additionally, Cu can activate phosphotyrosine-binding protein mediator of ErbB2-driven cell motility 1, which triggers EMT by binding to insulin receptor substrate 1 and activating downstream PI3K-AKT signaling pathway (Sorokin and Chen, 2013; Vitaliti et al., 2022). Furthermore, copper has been reported to regulate intracellular levels of cyclic adenosine monophosphate (cAMP) by binding to and inhibiting cAMP-degrading phosphodiesterase 3B activity (Krishnamoorthy et al., 2016). The primary target of cAMP - protein kinase A - is believed to enhance cancer cell metastatic capacity (Tonucci et al., 2019). Recent findings indicate that ATOX1 is essential for the migration of breast cancer cells (Blockhuys et al., 2020). The autophagy pathway plays an essential role in cancer cell proliferation as it facilitates recycling metabolic waste for further energy supply or aids in evading apoptosis (Xie et al., 2023). Tsang et al. discovered a direct interaction between Cu and the autophagic kinases unc-51 like autophagy activating kinase 1 (ULK1) and ULK2, demonstrating that intracellular excessive Cu ions are associated with starvation-induced autophagy, thereby enhancing ULK1 kinase activity and promoting autophagic flux (Tsang et al., 2020). Collectively, elevated levels of Cu can modulate various proteins and signaling pathways involved in cancer progression.

## 4 Cuproptosis in gynecological cancers

Gynecological cancer is a significant threat to women’s health and life, encompassing CC, OC, and UCEC. Recent advancements in next-generation sequencing technology and bioinformatic analysis methods have led to the discovery of numerous cuproptosis-related genes (CRGs) and cuproptosis-related long non-coding RNAs (CRLs) in patients with gynecological cancers. Utilizing these CRGs and CRLs, researchers have developed prognostic prediction models for patients with gynecological cancers through LASSO algorithms and multivariate regression analysis. The samples are stratified into high-risk and low-risk groups based on the risk score that exhibits a negative correlation with disease prognosis. Subsequently, they performed functional analyses of differentially expressed genes between high-risk and low-risk groups to identify potential biomarkers and therapeutic targets associated with cuproptosis-related signatures in gynecological cancers. Immunotherapy, such as anti-PD-1/PD-L1 antibodies represents an important milestone in gynecological cancer treatment. Nevertheless, the low response rate observed in unselected patients and the tendency of therapeutic resistance continue to pose significant challenges to their clinical application (Li T. et al., 2023). Thus, the potential benefits of immunotherapy for patients with different risks were also explored. Additionally, several studies suggest that copper ionophores such as elesclomol and disulfiram (DSF) may hold promise as treatments for gynecological malignancies.

### 4.1 Construction of risk models and functional analysis using CRGs/CRLs

#### 4.1.1 CC

CC is a prevalent tumor that poses a significant threat to the physical and mental wellbeing of women. To date, several studies have identified CRGs or CRLs associated with CC prognosis, developed prognostic models, and elucidated potential mechanisms influencing prognosis and therapeutic strategies through functional analysis of distinct risk groups (Table 1). Lei et al. (2022) investigated the differential expression levels of 13 known CRGs in cervical cancer samples and constructed a prognostic prediction model incorporating seven CRGs. Their findings revealed that DBT, FDX1, LIPT1, and PDHA1 acted as positive predictors for survival in patients with CC, while ATP7A, DLAT, and GCSH were negative predictors. Notably, LIPT1 and PDHA1 were found to positively regulate cuproptosis whereas ATP7A inhibited cuproptosis by reducing intracellular Cu levels (Tsvetkov et al., 2019; Tsvetkov et al., 2022), suggesting reduced susceptibility to cuproptosis in the high-risk group (Lei et al., 2022). Among these CRGs, FDX1 has been reported to impact the prognosis of lung adenocarcinoma by modulating glucose metabolism, fatty acid oxidation, and amino acid metabolism without affecting tumor cell growth rate apoptosis or abnormal cell cycle distribution (Zhang Z. et al., 2021). Furthermore, ATP7A has been implicated in poor survival outcomes across various cancer types due to its role in promoting tumorigenesis metastasis and conferring platinum drug resistance (Samimi et al., 2003; Shanbhag et al., 2019; Yu et al., 2021). Moreover, Kang et al. (2024) discovered that SFT2D1, a cuproptosis-related angiogenesis gene, exhibited high expression in CC and displayed a positive correlation with microvascular density. Knockdown of SFT2D1 significantly inhibited the proliferation, migration, and invasiveness of CC cells. Meanwhile, several significant CRLs have been validated to be included in risk prediction models. LINC01305 has been shown to promote the progression of CC cells and is associated with a lower survival rate through its interaction with the RNA-binding protein KHSRP (Huang et al., 2021). Liu et al. (2022b)’s study revealed that AL441992.1 is a protective factor in CC, which is consistent with previous research conducted by Chen et al. (2020). In CC, SOX21-AS1 exhibits hypomethylation and plays a role in cell proliferation, migration, invasion, and EMT progress (Zhang et al., 2019; Du et al., 2021). ATP2A1-AS1 has been confirmed as a prognostic biomarker for CC due to its involvement in autophagy-related processes (Feng et al., 2021). Additionally, Liu et al. (2023b) was the first to discover that CNNM3-DT was differentially expressed between radiosensitive and non-radiosensitive groups, suggesting it may serve as a potential target for treatment.

**TABLE 1:** CRGs/CRLs in the risk model and results of functional analysis between two risk groups in CC.

Author and publication year	CRGs/CRLs	Important GO terms	Important KEGG pathways	Differences in immune function
Lei et al. (2022)	ATP7A, DBT, DLAT, FDX1, GCSH, LIPT1 PDHA1	The signaling receptor activity, receptor ligand activity, extracellular matrix structural constituent	Focal adhesion, extracellular matrix receptor interaction	NK cells, aDCs, CD8^+^ T cells, pDCs
Liu et al. (2022b)	AL441992.1, LINC01305, AL354833.2, CNNM3-DT, SCAT2, AL354733, AC009902	Humoral immune response, leukocyte-mediated immunity, lymphocyte-mediated immunity	Cytokine–cytokine receptor interaction, cell adhesion molecules, oxidative phosphorylation	aDCs, B cells, CD8^+^ T cells, NK cells, pDCs, Tfh cells, and TILs; APC co-inhibition, checkpoints, HLA, T cell co-inhibition, T cell co-stimulation
Wang and Xu (2022)	AJ003147.1, AC096992.2, SOX21-AS1, AL049869.2, CNNM3-DT, ARHGAP31-AS1	T cell activation, lymphocyte differentiation, T cell receptor complex	Cytokine-cytokine receptor interaction, T cell receptor signaling pathway	Cytolytic activity, parainflammation, T cell co-inhibition
Liu et al. (2023b)	AC063943.1, CDKN2B−AS1, CNNM3–DT	Ligand-receptor activity, activator receptor signaling activity	MAPK signaling pathways, the interaction between cytokine receptors and the cytokines	APC co-stimulation, CCR, parainflammation, APC co-inhibition, checkpoint, MHC class-I, and T-cell co-inhibition
Zhou et al. (2023)	AC002128.2, AC009237.14, AC002563.1, AC048337.1, AC145423.1, AL117336.1, AP001542.3, ATP2A1-AS1, LINC00426		Th1 and Th2 cell differentiation, Th17 cell differentiation, T cell receptor signaling pathway, cytokine–cytokine receptor interaction, natural killer cell-mediated cytotoxicity, PD-L1 expression, PD-1 checkpoint pathway in cancer	Activated dendritic cell, CD56 bright natural killer cell, central memory CD4 T cell, effector memory CD8 T cell, eosinophil, γ delta T cell

CRGs, cuproptosis-related genes; CRLs, cuproptosis-related long-noncoding RNAs; CC, cervical cancer; GO, gene ontology; KEGG, kyoto encyclopedia of genes and genomes.

The results of Kyoto Encyclopedia of Genes and Genomes (KEGG) functional analysis revealed enrichment of immune-related pathways, including cytokine–cytokine receptor interaction, T cell receptor signaling pathway, Th1 and Th2 cell differentiation, as well as PD-L1 expression and PD-1 checkpoint pathway in cancer between two risk groups. These findings suggest that CRLs may mediate immune-related processes and ultimately impact the progression of CC in different risk groups (Liu et al., 2022b; Wang and Xu, 2022; Zhou et al., 2023). Furthermore, KEGG analysis demonstrated that differentially expressed CRGs were enriched in focal adhesion and extracellular matrix receptor interaction pathways closely associated with invasion and metastasis processes in cancer cells. This indicates a potential relationship between cuproptosis and CC invasion (Lei et al., 2022). Given the strong association between CRLs and immune regulation along with the widespread use of immunotherapy for CC treatment, an immune function analysis was conducted. Significant differences were observed in various immune cell types and functions between two risk groups. For instance, Liu et al. (2023b) reported significantly lower expression levels of CD8^+^ T-cells in the high-risk group, which is known to contribute to cancer progression through exhaustion mechanisms (Dolina et al., 2021). Additionally, key immune checkpoint genes exhibited higher expression levels in the low-risk group including PD-1, CTLA4, LAG-3, and TIGIT suggesting that immunotherapy such as immune checkpoint inhibitors (ICI) may be more suitable for patients with low-risk (Liu et al., 2022b). These conclusions provide novel insights into CC progression mechanisms as well as treatment strategies tailored to patients with different risk.

#### 4.1.2 OC

OC is the most lethal malignant gynecological tumor, with 5-year survival rates below 45% (Webb and Jordan, 2017). We identified four articles that elucidated the construction of prognostic prediction models and revealed significant differences in signal pathways and immune functions between high- and low-risk subgroups (Table 2). Among the candidate CRGs/CRLs involved in constructing the prediction model, functional experiments confirmed that WASF2 was associated with cuproptotic resistance, promoting cancer cell proliferation and platinum resistance. Moreover, its expression level showed a negative correlation with prognosis in OC patients (Wang et al., 2023). The lncRNA ZFHX4-AS1 was identified as a prognostic biomarker involved in cell proliferation, metabolism, infiltration, and distribution of tumor-infiltrating immune cells in OC. Its over-expression was significantly associated with poor overall survival and progression-free survival (PFS) (Wang et al., 2022). Additionally, AP001372.2 was reported as a novel biomarker for predicting prognosis in head and neck squamous cell cancer (Liu et al., 2022a). Liu et al. (2023a) found that the differentially expressed genes were enriched in extracellular matrix (ECM) related signaling pathways and biological processes in Gene Ontology (GO) and KEGG analysis. The ECM, which plays role in the transmission of information between cells, is remodeled by the uncontrolled growth of cells in cancer (Girigoswami et al., 2021). A recent review highlighted the pivotal involvement of ECM in serous OC development and progression including initiation at precursor lesions within fallopian tube fimbriae, metastatic progression, and drug resistance development (Brown et al., 2023). Moreover, significant differences in immune function analysis were observed among different risk groups, including immunomodulatory pathways, immune cell types, and immune cell infiltration. Zhang J. et al. (2022) found a negative association between the risk score and the immune score, activated immune inflammatory cells, the number of immune-related pathways, and particularly the immune cell infiltration. This suggests that patients in the high-risk group may experience immunosuppression. Liu et al. (2023a) reached a similar conclusion as they identified abundant Tregs in the tumor microenvironment of high-risk patients. Tregs have been reported to play a role in suppressing antitumor immune responses and are associated with poor survival rates (Göschl et al., 2019). The high-risk group also exhibited enrichment of pathways involved in promoting cancer progression such as WNT β-catenin signaling and EMT receptor interaction (Li et al., 2024). Meanwhile, patients in the low-risk group showed high expression levels of molecules related to immune checkpoints such as PDL1 and CTLA4 (Zhang J. et al., 2022; Li et al., 2024). In contrary, Liu et al. (2023a) noted overexpression of PD1, CTLA4, PD-L1, and HAVCR2 in patients from the high-risk group. Therefore, ICI treatment for OC patients should be individualized.

**TABLE 2:** CRGs/CRLs in the risk model and results of functional analysis between two risk groups in OC.

Author and publication year	CRGs/CRLs	Functional analysis	Differences in immune function
Zhang J. et al. (2022)	ZNF146, UPF1, TLE1, TEAD1, RALGAPB, PSMB9, PLEKHH1, LRRN2, KIAA0100, GTSE1, GPT2, DHRS13, AMMECR1	GSVA: TGF-beta signal, Notch signal, antigen processing	Activated B cell, activated CD8 T cell, activated dendritic cell, CD56 bright natural killer cell, gamma delta T cell, immature B cell, MDSC, monocyte, type 17 T helper cell
Liu et al. (2023a)	AP004609.3, AP003392.3, AP001372.2, AC021851.1	GO: extracellular matrix, extracellular structure, external encapsulating structure organization KEGG: extracellular matrix organization, extracellular structure organization, external encapsulating structure organization	Macrophages, T cells, resting NK cells; type II IFN response, CCR, APC co-inhibition, para inflammation, T cell costimulation, T cell co-inhibition and check-point; infiltration proportions of B cells, CD8 T cells, DCs, macrophages, neutrophils, Treg, T helper cells
Wang et al. (2023)	TIMM8B, COX8A, SSR4, HIGD2A, WASF2, PRDX5, CLDN4		CD4^+^ T memory cells, CD8^+^ naive T cells, CD8^+^ T cells, cDCS, aDCs, chondrocytes, mast cells, pericytes; the abundance of eosinophils, immature B cells, mast cells, NK cells, pDCs, regulatory T cells, T follicular helper cells, and Th1 cells
Li et al. (2024)	LIN C00189 LINC00861 ZFHX4-AS1 RPS6KA2 -IT1, LIN C00582 C9orf106 DEPDC1AS1 LINC01556 LEMD1AS1 TYMSOS	KEGG: Hedgehog signaling pathway, calcium signaling pathway, WNT signaling pathway, ECM receptor interaction, focal adhesion	Plasma cells, CD8 + T cells, helper follicular T cells, M1 macrophages, dendritic resting cells, M2 macrophages; interferon gamma response, allograft rejection, interferon alpha response, IL6-JAK-STAT signaling

CRGs, cuproptosis-related genes; CRLs, cuproptosis-related long-noncoding RNAs; OC, ovarian cancer; GSVA, gene set variation analysis; GO, gene ontology; KEGG, kyoto encyclopedia of genes and genomes.

#### 4.1.3 UCEC

UCEC, which originates from the endometrium, is a prevalent gynecological cancer worldwide. Table 3 summarizes the CRGs/CRLs in prognosis prediction models and the results of various functional analyses. Chen (2022) discovered that CDKN2A is overexpressed in UCEC cells and proposed that the regulatory axis lncRNA XIST/miR-125a-5p/CDKN2A plays a critical role in UCEC progression. Other studies have also reported CDKN2A as a prognostic biomarker in UCEC (Zhang et al., 2020; Lin et al., 2023). The CRG PC has been validated to be significantly upregulated in some UCEC cell lines (Lin et al., 2023) and has been shown to be essential for cell proliferation and progression in various malignancies (Sellers et al., 2015; Kiesel et al., 2021). lncRNA AC084117.1, associated with glutaminase, has been identified as a risk factor, and its silence significantly inhibits the proliferation and migration of UCEC cells (Cai et al., 2023). Another risk factor, BX322234.1, was found to be negatively correlated with prognosis in UCEC patients (Li B. et al., 2023), consistent with previous studies (Wang et al., 2021; Huo et al., 2022).

**TABLE 3:** CRGs/CRLs in the risk model and results of functional analysis between two risk groups in UCEC.

Author and publication year	Names of CRGs/CRLs	Functional analysis	Differences in immune function
Chen (2022)	CDKN2A, GLS LIPT1		
Cai et al. (2023)	AC084117.1, AC090617.5, FARSA-AS1, AC011479.2, Z99572.1, AC004466.2, AC008966.2		The type I and type II interferon responses, infiltration of CD8^+^ T cells; the TMB level
Hu et al. (2023)	AC073046.1, AC108479.1, ASH1L-AS1, LINC01644 AL132639.2, SBF2-AS1	GO: neuropeptide hormone activity, extracellular structure organization and extracellular matrix organization, anchored component of the membrane term	IFN, cytolytic activity inflammation promoting, T cell co-inhibition, CCR, check-point, T cell co-stimulation, MHC, parainflammation, type I IFN; the TMB level; the activity of checkpoint and CCR
Jiang et al. (2023)	LINC01545, AC02620.2, NRAV, AL450384.1, AC079466.2, AC090617.5	GO: microtubule- based movement, cilium organization, cilium assembly, cilium movement KEGG: neurodegeneration-multiple diseases, muscular atrophy-lateral sclerosis, neuroactive ligand receptor interaction	Activated CD8^+^ T cells, eosinophils, immature dendritic cells, MDSC, monocytes; type II IFN response, cytolytic activity, T cell co-stimulation, HLA; CD8 + T cell enrichment
Li B. et al. (2023)	AC093382.1, AL445985.1, AC079466.2, AL359962.3, AC060780.1, AC093157.2, AL592166.1, AC006230.1, AL078644.1, AC010201.3, BX322234.1, AC022467.1, AC007014.2	GO: cilium movement, microtubule-based movement, the motile cilium, cytoplasmic region, axoneme, tubulin binding, cytoskeletal motor activity, microtubule motor activity KEGG: pathways related to amyotrophic lateral sclerosis, tyrosine metabolism, cAMP signaling, vitamin digestion and absorption	The TMB level; response to IFN–I, MHC class I, APC costimulation, APC co-inhibition, type II IFN, parainflammation
Lin et al. (2023)	GLS, CDKN2A, PC, SUCLG1	IL-1 signaling pathway, cellular response to cytokine stimulus	The proportion of MSI-H; macrophages, B cells, aDC; eosinophils, iDC, mast cells, NK CD56bright cells, NK cells, pDC, T cells, Th17 cells
Pang et al. (2023)	ATF5, FBXO46, P2RX4, SMARCD3 DAPK3, C1orf53		Four existing immune subtypes; active dendritic cells, B cells naive, resting T cells CD4 memory, T cells follicular helper, regulatory T cells, resting dendritic cells, T cells CD8, plasma cells; the TMB level
Qi et al. (2023)	AC007552.2, AC090617.5 AC026202.2 AC073046.1 CDKN2A-DT	GO: the movement of microtubules, antigen binding, cytoplasmic region KEGG: PI3K-AKT signaling pathway, MAPK signaling pathway, neurodegenerative pathways	The TMB level; T-cell costimulatory, cytolytic activity

CRGs, cuproptosis-related genes; CRLs, cuproptosis-related long-noncoding RNAs; UCEC, uterine corpus endometrial carcinoma; GO, gene ontology; KEGG, Kyoto encyclopedia of genes and genomes; TMB, tumor mutational burden; MSI-H, high microsatellite instability.

The results of GO, KEGG, and immune functional analysis revealed significant differences between the low-risk and high-risk groups. Hu et al. (2023) identified neuropeptide hormone activity as the top-ranked term in GO analysis, with extracellular structure organization and extracellular matrix organization also enriched. These findings suggest an association between UCEC prognosis and cellular recognition and hormone modulation (Hu et al., 2023). In terms of KEGG analysis, several critical signaling pathways in cancer research were enriched, including cAMP signaling, PI3K-AKT signaling pathway, and MAPK signaling pathway (Li B. et al., 2023; Qi et al., 2023). The activated cAMP/PKA signaling pathway has been shown to inhibit proliferation, invasion, migration, and growth of UCEC cells in mice (Li et al., 2022). Aberrant activation of the PI3K-AKT signaling pathway has been reported in various malignancies including UCEC (Noorolyai et al., 2019; Roncolato et al., 2019). In UCEC, excessive estrogen can activate the PI3K-AKT pathway to promote cell proliferation (Qu et al., 2015), while inhibitors targeting this pathway have demonstrated potential for suppressing progestin-resistant cancer cell proliferation through autophagy promotion (Liu et al., 2017). Furthermore, numerous studies have focused on exploring potential therapies by targeting the PI3K-AKT pathway in UCEC (Roncolato et al., 2019; Lin et al., 2022; Liang et al., 2023). The MAPK pathway, consisting of a cascade of three kinases, is known to respond to various physiological signals such as hormones, cytokines, and growth factors (Lee et al., 2020). It has been found to play a role in the migration and invasion of cancer cells in UCEC (Zhang F. et al., 2021). Additionally, immune analysis revealed significant differences in immune cell types, immune function, and immune cell infiltration between the high-risk and low-risk groups. The low-risk group exhibited higher checkpoint and CCR activity, higher tumor mutational burden (TMB), as well as a higher proportion of high microsatellite instability compared to the high-risk group (Hu et al., 2023; Li B. et al., 2023; Lin et al., 2023). Theoretically speaking, a higher TMB leads to more neo-antigens which increases the chances for T cell recognition and improves outcomes in clinical ICI treatment (Jardim et al., 2021). Consequently, patients in the low-risk group may potentially benefit more from immunotherapy.

### 4.2 Application of copper ionophores in gynecological cancers

Copper ionophores are lipid-soluble molecules capable of increasing intracellular Cu levels and inducing cuproptosis by reversibly binding with Cu (Tsvetkov et al., 2022). Elesclomol and DSF, as prominent copper ionophore drugs, have been shown to play a crucial role in cancer therapy (Oliveri, 2022). For instance, a phase III trial demonstrated that the combination of elesclomol and paclitaxel significantly extended median PFS compared to paclitaxel alone in advanced melanoma patients with normal baseline lactate dehydrogenase levels (O’Day et al., 2013). DSF, an inhibitor of aldehyde dehydrogenase, has been suggested to confer protective effects against prostate and breast cancer (Askgaard et al., 2014).

Several studies have demonstrated the potential application of elesclomol and DSF in the therapy of gynecological cancers. In CC cell lines, DSF showed cytotoxicity in a Cu-dependent manner and this cytotoxic effect was mediated by apoptosis and autophagy simultaneously. Additionally, the DSF/Cu complex was found to reduce cisplatin resistance by targeting cancer stem cell-like LGR5(+) cells (Cao et al., 2022; Zhang W. et al., 2022). Furthermore, Zheng et al. (2023) revealed that DSF could bind to HSP90A to inhibit tumor growth and metastasis through the HSP90A/NDRG1/β-catenin pathway in CC cells. Combination treatment with DSF and Cu significantly reduced tumor volume and improved survival rate in a murine OC xenograft model (Gan et al., 2023). Moreover, DSF was shown to enhance drug sensitivity of poly (ADP-ribose) polymerases inhibitors (PARPis), an important targeted drug for OC treatment (Tang et al., 2023). Additionally, both elesclomol and DSF increased cell death when combined with carboplatin treatment compared to carboplatin alone in OC cells. The combination of DSF and cisplatin also enhanced apoptosis of cancer cells, suggesting that Cu ionophores could augment the anti-tumor effect of platinum drugs (Harrington et al., 2020; Yuan et al., 2023). In Ishikawa cells (endometrial cancer cells), the combined application of DSF and a novel Cu-cysteamine compound has been shown to promote apoptosis and exert potent anti-tumor effects by inducing mitochondrial impairment (Yang et al., 2024). Overall, DSF and elesclomol not only have efficacy against gynecological malignancies, but also show the potential to enhance the drug sensitivity of other anti-tumor chemotherapy drugs or targeted therapies.

## 5 Conclusion

The essential micronutrient Cu serves as a critical catalytic cofactor in various biological processes and maintains homeostasis. Excessive intracellular Cu is associated with the development of diseases such as Wilson disease. Cu from dietary sources or water is absorbed in the small intestine and transported into the bloodstream by ATP7A. The liver plays a crucial role in regulating systemic Cu homeostasis by storing Cu, excreting excess Cu into bile ducts, and releasing Cu into the blood for redistribution. Within cells, Cu is either stored bound to MTs or transported within the cytoplasm by copper chaperones. It can target SOD1 through CCS to detoxify ROS, bind to ATOX1 for delivery to secretory and biosynthetic pathways, or be transported to CCO within mitochondria for oxidative phosphorylation and mitochondrial function via COX17. In March 2022, Tsvetkov et al. elucidated a novel mechanism of cell death induced by Cu called cuproptosis, which differs from other programmed cell death mechanisms. Copper ions (Cu+) can target lipoylated enzymes in the TCA cycle, subsequently inducing oligomerization of lipoylated DLAT, loss of Fe-S clusters, and induction of HSP70 expression leading to acute proteotoxic stress-induced cell death.

The levels of Cu are elevated in both serum and tumor tissues in numerous malignancies. Recent studies have demonstrated that excessive Cu can promote cancer progression by enhancing tumor growth, proliferation, angiogenesis, and metastasis through various signaling pathways, such as the RAS/RAF/MEK/ERK pathway and the PI3K-AKT signaling pathway. ATOX1, a copper chaperone, is considered to function as a Cu-dependent transcription factor promoting inflammatory neovascularization. Additionally, copper has been reported to be involved in regulating vascular formation and infiltration, activating tumor microenvironment remodeling, inducing EMT, and participating in autophagy processes that contribute to cancer progression.

CC, OC, and UCEC are prevalent gynecological malignancies that have inflicted suffering and claimed the lives of women worldwide. Recent studies have identified specific CRGs or CRLs as prognostic markers for these cancers, aiming to elucidate the role of cuproptosis in their development by constructing risk prediction models. Multiple pathways, including immune-related pathways, WNT β-catenin signaling, cAMP signaling, PI3K-AKT signaling, and MAPK signaling pathway, have been implicated in the progression of these cancers. Furthermore, significant differences in immune function were observed between high-risk and low-risk groups, suggesting that immune therapy may be more effective in the latter group. Additionally, copper ionophores such as elesclomol and DSF hold promise for treating gynecological malignancies due to their direct effects on tumor cells as well as their ability to enhance sensitivity to other anti-tumor drugs like PARPis and platinum-based agents.

## Figures and Tables

**Table:** 

**ATOX1**	antioxidant-1 protein
**ATP7A**	ATPase copper transporting alpha
**ATP7B**	ATPase copper transporting beta
**cAMP**	cyclic adenosine monophosphate
**CC**	cervical cancer
**CCO**	cytochrome c oxidase
**CCS**	copper chaperone for superoxide dismutase
**CKGs**	cuproptosis key genes
**COX17**	cytochrome oxidase 17 protein
**CTR1**	copper transporter 1
**Cu**	copper
**Cu-ATPase**	Cu-transporting ATPase
**DLAT**	dihydrolipoamide S-acetyltransferase
**DLD**	dihydrolipoamide dehydrogenase
**DMT1**	divalent metal transporter 1
**DSF**	disulfiram
**FDX1**	ferredoxin 1
**GO**	Gene Ontology
**GSH**	glutathione
**HIFs**	hypoxia inducible factors
**HSP70**	heat shock protein 70
**ICI**	immune checkpoint inhibitors
**KEGG**	Kyoto Encyclopedia of Genes and Genomes
**LIAS**	lipoyl synthase
**LIPT1**	lipolytransferase1
**MEK1/2**	mitogen-activated protein kinase 1/2
**MTs**	metallothioneins
**OC**	ovarian cancer
**PARPis**	poly (ADP-ribose) polymerases inhibitors
**PDH**	pyruvate dehydrogenase
**PDHA1**	pyruvate dehydrogenase E1 subunit alpha 1
**PDHB**	pyruvate dehydrogenase E1 subunit beta
**PFS**	progression-free survival
**ROS**	reactive oxygen species
**SLC31**	solute carrier 31
**SOD1**	superoxide dismutase 1
**TCA**	tricarboxylic acid
**TMB**	tumor mutational burden
**UCEC**	uterine corpus endometrial carcinoma
**ULK1**	unc-51 like autophagy activating kinase 1
**VEGF**	vascular endothelial growth factor
